# A Case of Traumatic Tetanus Successfully Treated with the Calabar Bean

**Published:** 1868-06-15

**Authors:** A. J. Baxter

**Affiliations:** Chicago, Ill.


### A CASE OF TRAUMATIC TETANUS SUCCESSFULLY TREATED WITH THE CALABAR BEAN.

BY A. J. BAXTER, M.D., CHICAGO, ILL.

*Reported by Dr. P. Curran.*

From time immemorial, traumatic tetanus, once fully established, has been considered fatal. The ancient authorities declare it to be so, and unfortunately the experience of all times has but too truly confirmed the statement — all treatment up to the present being considered empirical.

### Case.

Benjamin Cleves, a member of Dearborn Light Artillery, whilst engaged in firing a salute, at about 4.30 A.M., on the 4th of July, 1867, had the front right forearm terribly mangled by the premature discharge of the piece. He was removed to a fire engine house near by, where Dr. Baxter saw him in less than thirty minutes after the accident occurred. His face and eyes were full of powder, the muscles of the forearm were very much lacerated, in some parts hanging in shreds, and the ends of some of the fingers blown off. Immediately below the bend of the arm there was an immense tumor, very tense, yet giving an indistinct sense of fluctuation, which was thought to contain extravasated blood. There was no pulsation in either the radial or ulnar arteries. The surface of the hand and arm was cold. Large splinters of wood, which seemed to be fragments of the ramrod, were imbedded throughout the arm. It having been determined to save the arm, if possible, warm water dressings were applied. He was taken to his hotel.

6.30 A.M.— He complained very much of pain. Slight circulation in radial and ulnar. Warm water dressings continued. Beef tea, one half grain of *morphine* every two or three hours, if necessary. From this time till the morning of the 8th, every thing went along well, when Dr. B. was sent for in great haste, and informed that he was bleeding terribly from the tumor below the bend of the arm. On the doctor's arrival the hæmorrhage had ceased, and the tumor had disappeared. The blood was described as being black, thin, and offensive.

9th.—Slough had all separated; granulation looked well. Warm water dressings discontinued. Arm dressed with *Cerat. zinci carb.; Morphine* powder at night.

14*th.*—Complained of general uneasiness, or soreness; granulations looked pale and shrunken; pulse 110. Ordered California port wine *ad libitum;* beef essence and *cerat. zinci* discontinued; warm water dressings applied.

15*th.*—Did not rest well last night; still complained of soreness; countenance anxious; pulse 130; has had several spasms of the flexor muscles of the forearm, which flex the wrist forcibly on the forearm, causing much pain, which is referred to the back of the wrist. Half grain of *Morphine* every two or three hours, according to circumstances.

6 P.M.—Spasms more frequent and severer, but confined to the injured arm. Was questioned closely as to the mobility of jaws, neck, and back. Thought to be all right by having them worked vigorously. Threw twenty drops of Magendie's solution of *Morphine* under the skin over the wrist, being the point where the greatest amount of pain was complained of, with the effect of mitigating the spasms, both as to frequency and severity, for several hours. 11 P.M.—Spasms as bad as ever. One grain of *Morphia* ordered every two hours; 40 drops of Magendie's solution under the skin over the wrist.

16*th*, 8 A.M.—Worse generally. The *Morphine* does not produce the slightest impression on the spasms, which occur as often as every five minutes, causing a great deal of pain in the wrist and hand. Ordered *Gelsemin* gr. ii. every two hours; all the wine and nourishment he could take. 1 P.M.—Spasms as frequent as ever. *Gelsemin* powder every hour. It may be stated here, that henceforward, until he was quite well, that stimulants, in the form of California port wine and beef tea, were given to the fullest extent. Mention will only be made of the medication. 6 P.M.—Very much prostrated from the effects of the *Gelsemin*; pupils dilated; vision disordered; complained of heaviness of the eyes, etc., etc.; pulse 150; spasms as frequent as ever, and more violent. Patient said he must have morphine. Ordered *Extr. Cannabis Indicæ*, gr. ii. every two hours; gr. 1½ *Morphiæ acetat.* as required to blunt the sensibility to the pain of the spasms. Threw 40 drops of Magendie's solution into the arm.

17*th*, 8 A.M.—The spasms are becoming general — worse on the side opposite the injury. Complained of soreness and stiffness about the jaws and neck, without having his attention specially called to them; thought he could not open his jaws as wide as usual. Dr. Baxter, finding the ordinary remedies prove unavailing, consulted with the leading physicians of the city, and all came to the conclusion that without amputation there could be no chance of recovery. Dr. B. amputated the arm near the shoulder; the operation partially relieved the spasms; however, the disease was making headway, when *Calabar bean* was suggested as worthy of trial. It was brought into requisition, but for some two days seemed to produce no decided effect.

Having accompanied Dr. Baxter on a visit to the patient, I found him in a worse condition than when I had seen him on a former occasion. The muscles of the neck were swollen, hard, and contracted; his jaw was nearly shut by trismus; he complained of his tongue being swollen so much as to fill up his mouth; his countenance bore a turbid, rather than an anxious appearance; his eyes seemed wearied; his friends were greatly depressed on account of his state.

Dr. Baxter requested me to remain with the patient during the night. He gave directions to have fifteen drops of the extract of *Calabar bean* administered every two hours, with *Morphine* powders of a grain each to be given in quantities sufficient to produce sleep. The administration of the *Morphine* was necessarily left to my own discretion.

About eight o'clock in the evening he was much inclined to sleep — trying to fall off into a slumber every four or five minutes, but roused from the attempt each time by a spasm of the stump of the amputated arm — the twitchings were emprosthotonic. After each spasm he would rub the muscles of the face and forehead with his hand; occasionally he would rub his eyes. Dr. Baxter injected the stump of the arm with Magendie's solution of *Morphia* before he left. I administered the *Calabar bean* according to directions, and gave the *Morphine* from time to time, endeavoring to produce its narcotic effect. At ten o'clock there was no visible change, the patient suffering pain, and very restless, requiring to have his position changed every fifteen minutes. His attendants complied with his wishes very sedulously. I shortened the intervals in administering the *Morphine*, and by twelve o'clock he seemed to be easier. I watched him very closely at this stage of the treatment, lest either the *Morphine* or the *Bean* should produce an effect greater than I desired. I found the pulse to be getting slower and fuller; he seemed to have less pain. I continued giving the *Morphine* and *Bean* regularly up to half past two, when a change for the better was visible. I now took his wrist into my hand, and held my fingers immovably on his pulse, that I might clearly understand the changes of the heart's action during the repose he was, as I supposed, about to take. In about a quarter of an hour, he, with much difficulty, really got to sleep. The spasms of the arm interrupted his slumber several times within the quarter of an hour; still sleep came on notwithstanding the twitchings. During the quarter of an hour preparatory to sleep, and the same amount of time during which he did sleep, the pulse exhibited strange phenomena. When going to sleep, a striking change in the pulsation took place. It became hard, wiry, and slow. At times it would cease for a moment, when a spasm would come on, then it would recover its action, becoming full and strong. This cessation of pulse before a spasm, and the quickness and fullness of its volume after a spasm, was invariable during the interval of distressful repose. It appeared that the nervous system could scarcely get to rest without imminent danger of a total cessation of the heart's action. I was convinced that if the difficulty of getting properly to sleep was but fairly overcome for five minutes' time, that the great trouble in tetanus would be over. There is a physiological difficulty at the bottom of the disease. The spirit — the active principle — has suffered a loss, which it refuses to have restored.

When the patient re-awakened, he appeared conscious of having obtained a benefit; for he said to those around him: "Now that I have slept, I think that the trouble is over." He became gradually better until five o'clock, when trismus had ceased, and the muscles of the neck became pliable. I continued the same treatment throughout, until half past five, when I left, till nine. I told the attendants to administer the *Bean* as usual, and to give the *Morphine* powders in half grain doses every hour till my return. At nine o'clock he was much improved. He had taken the equivalent of sixty grains of opium during the hours I was in attendance for the first time.

During the whole of that day he was very nearly free from trismus. The twitchings of the arm would recur each time he attempted to sleep.

That night, Dr. Baxter thought my services still required, lest any untoward symptom should be manifested before morning. I strictly followed instructions that night, which instructions were, to give as little *Morphine* as possible. I administered the *Bean*, but gave no *Morphine.* The patient was very restless during the night. He wanted to sit on the chair; to have his bed adjusted and re-adjusted, time and again. Towards four o'clock in the morning he was getting worse. His pulse was 120. The spasms of the arm and shoulder were unabated, but not violent. I left at five o'clock in the morning, promising to be back at seven. When I returned, I found that the spasms became violent during my absence, episthotonos having set in with severe pain, which the patient called an agony. This occurred about six o'clock.

They had sent for Dr. Baxter. As soon as I arrived, I immediately gave a grain and a half of *Morphine*. When Dr. Baxter came, I apprised him of the condition of the patient. The doctor ordered the *Calabar bean* to be given every hour, fifteen drops at a dose, and *Morphine* at discretion. I promised the patient that by twelve or one o'clock I would have him out of his sufferings. I determined to push the *Morphine* treatment to the utmost, and if there were anodyne and antispasmodic qualities in it, to produce them. I gave a grain and a half of *Morphine* every hour, also fifteen drops of the *Calabar bean* extract — there being a quarter of an hour between the administration of each. The treatment was severe, but the case was now becoming desperate. I watched the patient closely, and found that I was gaining but little ground, till ten o'clock, when a decided improvement was observable. Dr. Baxter came about this hour. He was a little surprised at the quantities of *Morphine* I was giving; nevertheless, the effect produced was but slight. I continued the same treatment till half past eleven, when the opisthotonos and pain ceased. The patient became greatly relieved, and turned cheerful. His countenance assumed a natural and lively aspect. He conversed freely with his friends. From seven till eleven and a half A.M., I gave him the equivalent of forty-two grains of opium, without producing its narcotic effect. It was Dr. Baxter's opinion that the *Morphine* and *Calabar bean* mutually aided each other. I thereafter gave him the remedies regularly in half doses, for the purpose of maintaining the effect produced. The twitchings of the arm, from being opisthotonic, became emprosthotonic. I did not attend the succeeding night, and do not know what was the course pursued.

However, on the evening of the following day, Dr. Baxter announced to me that the patient was getting worse. I suggested a return to the *Morphine* treatment in conjunction with the *Calabar bean*. On going to see the patient, we found him suffering severe pain in the arm, shoulder, and neck. He had retrograded somewhat. I remained that night, at Dr.

B.'s request. He injected Magendie's solution of *Morphia* under the skin of the arm, and ordered a cloth dipped in a decoction of tobacco to be applied to the wound of the stump. This application evidently assisted greatly in alleviating the pain. I did not spare the *Morphine.* In four hours and a half, the equivalent of fifty-four grains of opium were given, in conjunction with the *Calabar Bean*, fifteen drops every hour and a half. At 12.50 o'clock, pain ceased; at 1.15, the patient went to sleep. Thenceforward he improved steadily till six o'clock A.M., when I left. He was under the care of Dr. Baxter from that time forward, and has perfectly recovered. I regard this case as really a cure. So much so that I felt able, during the treatment, to control the spasms and pain, calculated the time required to subdue them, and announced beforehand the hour when both contraction and pain would cease. I regulated the treatment according to the violence of the attack. The pulse was my guide throughout.

The principle acted upon was this: to regard the effect of the remedies given, not the quantity. The amount of *Morphine* and *Calabar bean* administered was extraordinary, still the nervous system, in its state of great irritability, was able to withstand it. It goes to prove that in tetanus the ordinary rules for the use of potent drugs are of no avail. I meant to find if any quantity whatever could control the disease. It was controlled. The patient is now perfectly well. The cure was due to Dr. Baxter's judicious and courageous treatment.

The formula we used was devised in haste, you will remember.

Not having time to macerate the *Bean*, we used a larger portion of spirit than necessary, perhaps, to thoroughly deplete.

To get rid of the spirit, and reduce it easily to a definite measure, we displaced the spirit with *Glycerine*, removing the evaporating dish when it ceased to lose weight.

| | | |
|---|---|---|
| Powdered *Calabar Bean*, | . . | 150 grs. |
| *Rectified Spirits*, | . . . . | q. s. |
| *Glycerine*, | . . . . .. | ℥i. |

First moistened the finely powdered *Bean* with a little spirit, then packed it firmly in a small glass percolator, adding successive portions (two drams) of spirit, until two ounces had passed, then displaced the spirit with the *Glycerine* in a porcelain capsule, by evaporating slowly until it ceased to lose weight.

*Dose.*—♏ xij., equivalent to gr. iij, of the powdered *Bean*.

